# Left ventriculo-arterial coupling in a contemporary cohort of patients with wild-type transthyretin cardiac amyloidosis treated with tafamidis

**DOI:** 10.1007/s00392-025-02727-z

**Published:** 2025-11-03

**Authors:** Giuseppe Damiano Sanna, Valeria Anna Di Simone, Paolo Milani, Alessandro Fogliani, Roberta Mussinelli, Gianluigi Guida, Andrea Attanasio, Laura Obici, Marco Basset, Martina Nanci, Andrea Foli, Gavino Casu, Leonardo De Luca, Mario Nuvolone, Giampaolo Merlini, Stefano Perlini, Giovanni Palladini

**Affiliations:** 1 Department of Molecular Medicine, University of Pavia, Pavia, Italy; 2 Department of Internal Medicine, University of Pavia, Foundation IRCCS San Matteo, Amyloid Research and Treatment Center, Pavia, Italy; 3 Clinical and Interventional Cardiology, Sassari University Hospital, Sassari, Italy; 4 Division of Cardiology, Fondazione IRCCS Policlinico San Matteo, Pavia, Italy; 5 Clinical Cardiology, IRCCS Policlinico San Donato, Milan, Italy; 6 University of Pavia, Department of Internal Medicine, Viale Golgi, 19, Pavia, 27100 Italy

**Keywords:** Wild-type transthyretin cardiac amyloidosis, Arterial elastance, Ventricular elastance, Ventriculo-arterial coupling, Echocardiography

## Abstract

**Background:**

Wild-type transthyretin amyloid cardiomyopathy (ATTRwt-CM) is characterized by a labile equilibrium between preload and afterload. A tailored approach to supporting medical therapy based on noninvasive parameters able to describe the properties of both heart and systemic vasculature, and their interactions is required. However, data on ventriculo-arterial coupling (VAC) in ATTRwt-CM is lacking.

**Objectives:**

To describe ventriculo-arterial coupling (VAC) and its clinical correlates in a contemporary cohort of patients with ATTRwt-CM.

**Methods:**

The VAC, defined as the ratio between arterial (*E*_*a*_) and ventricular elastance (*E*_*es*_) was evaluated noninvasively using the single-beat algorithm based on arm cuff blood pressure, Doppler stroke volume, pre-ejection time and total ejection time.

**Results:**

The study included 114 patients treated with the transthyretin stabilizer tafamidis from a national referral centre (median age 79 years; 89% males). Median values were 1.48 (1.22–1.84) mmHg/ml for *E*_*a*_, 1.86 (1.49–2.29) mmHg/ml for *E*_*es*_, and 1.24 (0.96–1.58) for VAC. Patients with upper-tertile VAC showed worse clinical (National Amyloidosis Centre (NAC)/Mondor stage, *p* < 0.001), laboratory (NT-pro-B-type natriuretic peptide levels, *p* < 0.001), instrumental features (left ventricular ejection fraction and stroke volume, *p* = 0.0001 for both), and they received more intensive heart failure supportive therapies. The *E*_*a*_/*E*_*es*_ ratio, but not its single components, was associated with NT-proBNP levels. Finally, the *E*_*a*_/*E*_*es*_ ratio was an independent determinant of a high NAC/Mondor stage at both univariate (OR[95% CI]:15.39[3.51–67.35], *p* < 0.001) and multivariate (OR[95% CI]:11.26[1.98–63.81], *p* = 0.006) logistic regression analyses.

**Conclusion:**

In ATTRwt-CM patients, arterial and ventricular elastances and VAC are independent predictors of worse clinical status and more advanced disease stage.

**Graphical Abstract:**

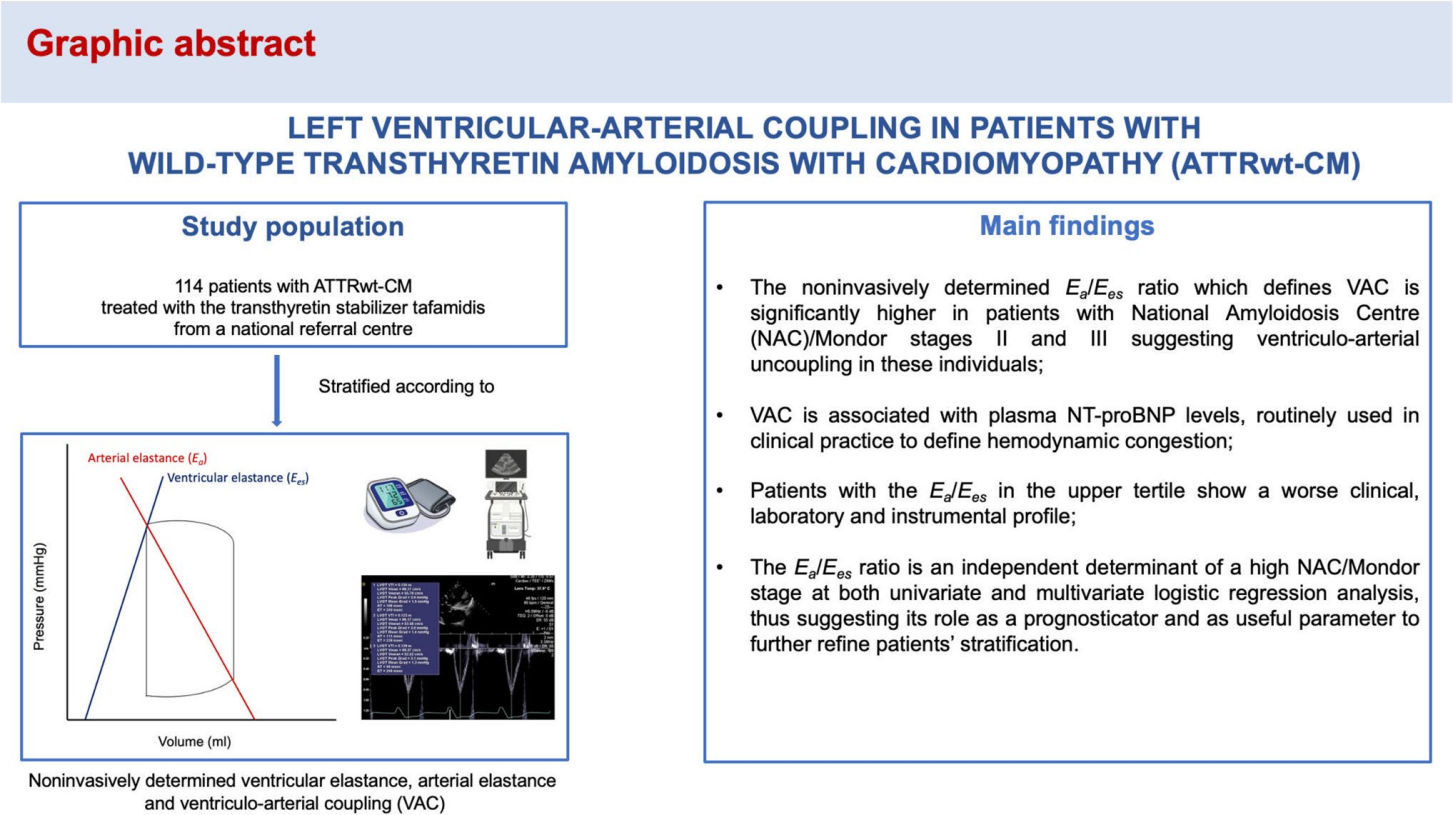

**Supplementary Information:**

The online version contains supplementary material available at 10.1007/s00392-025-02727-z.

## Introduction

Wild-type transthyretin amyloidosis (ATTRwt) is the most common amyloidosis involving the heart. It is caused by the deposition of wild-type transthyretin, and current treatment is aimed at reducing the supply of the amyloid precursor, by gene silencing, or at stabilizing it [1]. Clinical features include congestive heart failure (HF), arrhythmias and conduction disturbances, syncopal episodes and sudden cardiac death. ATTRwt presents as a restrictive cardiomyopathy with complex pathophysiology characterized by a labile equilibrium between preload and afterload, and data on efficacy of conventional HF therapies in this setting is still scarce [1, 2]. Hemodynamics should significantly vary among patients; therefore, the idea of a tailored supporting medical therapy in patients with ATTRwt cardiomyopathy (ATTRwt-CM) based on noninvasive parameters able to accurately describe the properties of both heart (i.e., left ventricle—LV) and systemic vasculature, as well as their interaction (i.e., ventriculo-arterial coupling) is intriguing. During the 1970 s of the twentieth century Suga and Sagawa, starting from the pressure–volume loops proposed the use of the end-systolic pressure–volume relation (ESPVR) and its slope (ventricular elastance—*E*_*es*_) to assess LV contractility [3–6]. A few years later, in 1983, Sunagawa introduced the concept of arterial elastance (*E*_*a*_), as an integrated index of afterload related to the characteristics of the arterial tree [7, 8]. In optimal hemodynamic states heart and vessels are “coupled”, and the *E*_*a*_*/E*_*es*_ ratio is around 1. Significant variations of this ratio (due to marked changes of *E*_*a*_ or *E*_*es*_) result in ventriculo-arterial uncoupling [9, 10]. However, the need of serial invasive measurements of LV pressures and volumes recorder over a range of cardiac loading for *E*_*es*_ determination has hampered the broader application of this parameter in routine clinical practice. In 2001, Chen firstly described a non-invasive echocardiographic single-beat method to calculate *E*_*es*_ [11]. Since then, several reports have started appearing in which this noninvasive approach is used in different settings (mainly in HF patients), and dedicated apps have been developed to facilitate bedside evaluation by clinicians (e.g., in the intensive care units) [12–15](https://apps.apple.com>app>ielastance). However, data on ventriculo-arterial coupling (VAC) in patients with ATTRwt-CM is still lacking. The aims of the present study were: (1) To describe VAC (as based on a noninvasive echocardiographic approach) in contemporary patients with ATTRwt-CM already treated with the transthyretin (TTR) stabilizer tafamidis [16]; (2) To analyse the relationship between VAC and overall disease status and progression; and (3) To describe the potential differences in terms of prescription of conventional supporting HF medications across different levels of ventriculo-arterial uncoupling.

## Methods

### Study population

This is an observational cross-sectional study that included patients diagnosed with ATTRwt-CM already enrolled in the prospectively maintained database of the Amyloidosis Research and Treatment Center Fondazione IRCCS Policlinico San Matteo, Pavia (Italy). All patients gave written informed consent for the use of their clinical data for research, and the study was approved by the Institutional Ethics Committee (NCT04839003). The study was conducted in accordance with the Declaration of Helsinki.

Main inclusion criterion was an ascertained diagnosis of ATTRwt amyloidosis with cardiac involvement. In this contemporary cohort medical treatments included in all patients the transthyretin stabilizer tafamidis 61 mg o.d. [16] (median treatment duration 18 [interquartile range — IQR 12–24] months). Patients were considered eligible to tafamidis treatment only if they had a New York Heart Association (NYHA) functional class I or II, according to the limitations imposed by the national (Italian) Medicines Agency. HF supporting therapies were prescribed according to the international guidelines for the management of patients with HF, and the position statements on cardiac amyloidosis. There were no restrictions in terms of upper patients’ age, and all subjects > 18 years were considered eligible for the purposes of the study.

Main exclusion criterion was represented by a poor-quality echocardiographic examination. Since all patients of this contemporary cohort were treated with tafamidis, those with a NYHA class III or IV at previous outpatient follow-up visit were excluded from the study.

Only patients diagnosed and treated at Amyloidosis Research and Treatment Center in Pavia were considered eligible for the present study, in order to reduce the potential sources of bias.

### Cardiac ATTRwt amyloidosis assessment

The diagnosis of ATTRwt amyloidosis with cardiac involvement was established according to the international consensus criteria [17]. A non-biopsy proven diagnosis was deemed possible and established in patients with clinical and instrumental (e.g., echocardiogram, cardiac magnetic resonance) red flags for amyloid deposition along with cardiac uptake on ^99m^Tc-DPD (or ^99m^Tc-HMDP) scintigraphy (score 2 or 3 by Perugini scale), in the absence of monoclonal components [17, 18]. These were excluded by the concomitant negative serum and urine protein electrophoresis with immunofixation and serum free light chains ratio (FLCR) within the reference range according to Katzman et al. (i.e., 0.26–1.65) [19]. All patients with clinical and/or instrumental findings suggestive for amyloid cardiac involvement and a concomitant monoclonal protein were required to undergo an endomyocardial biopsy if less invasive approaches (i.e., fat pad aspirate or a minor salivary gland biopsy) were negative. All required biopsies were stained with Congo red and TTR deposits were typed by immune-electron microscopy and/or mass spectrometry. In all cases, DNA analysis was performed in order to exclude hereditary forms due to mutations in *TTR* and *APOA1* genes encoding transthyretin and apolipoprotein A-I [20].

### Clinical records, laboratory and instrumental features

A detailed medical history was collected for all the patients including: family history, features suggestive of systemic amyloidosis (e.g., the presence of carpal tunnel syndrome, lumbar spinal stenosis), comorbidities and cardiac symptoms. A detailed pharmacological evaluation including all the cardiovascular drug classes, active ingredients and their doses, was also collected due to their potential effects on VAC and its components.

Apart from NYHA functional class, patients were subdivided according to the NAC (National Amyloidosis Centre)/Mondor prognostic staging system as follows: stage I defined as N-terminal pro-B-type natriuretic peptide (NT-proBNP) < 3000 ng/l and estimated glomerular filtration rate (eGFR) > 45 ml/min/1.73 m^2^; stage III defined as NT-proBNP > 3000 ng/l and eGFR < 45/min/1.73 m^2^; stage II the remainder [21]. This was possible because on the same day of the scheduled follow-up visit in the outpatient clinic, all patients underwent a comprehensive laboratory evaluation including cardiac biomarkers (i.e., NT-proBNP [or BNP for those with advanced/end-stage renal disease] and high-sensitivity troponin I), renal function and TTR (i.e., prealbumin) serum levels.

Physical examination included anthropometric data (height, weight, body surface area — BSA) and vital signs (cuff blood pressure both in the supine and upright position — using an automatic digital monitor [OMRON® M2], oxygen saturation, heart rate).

Patients’ assessment included both 6-Minute Walk Test (6MWT) and Kansas City Cardiomyopathy Questionnaire (KCCQ).

A standard 12-lead ECG was recorded in all the study patients in the supine position using commercially available electrocardiographs at a paper speed of 25 mm/s and 10 mm/1 mV standardization. We considered low QRS voltages in case of a QRS amplitude ≤ 0.5 mV in all limb leads or ≤ 1 mV in all precordial leads [21]. The peripheral QRS score was also calculated, as previously described [22].

Echocardiographic examinations were performed by a single experienced and accredited operator (G.D.S.) using a ACUSON SC2000 Prime cardiovascular ultrasound system (SIEMENS Healthineers®) equipped with a 1.1–4.9 MHz transducer. All the study patients underwent a comprehensive transthoracic examination in the left-lateral decubitus position. Doppler tracings and two-dimensional images were obtained from parasternal long- and short-axis, apical and subcostal views. In the presence of irregular heart rhythm (e.g., atrial fibrillation—AF), multiple loops (at least five) and averaged measurements were obtained. Standard cardiac chamber quantification was performed in accordance with the current American Society of Echocardiography (ASE) and European Association of Cardiovascular Imaging (EACVI) recommendations [23]. LV volumes were calculated from apical four-chamber and two-chamber views. LV global longitudinal strain (GLS) values were obtained from apical three-chamber, four-chamber and two chamber views.

Stored images and loops were reviewed by the same operator who repeated all the measurements offline.

### Arterial elastance (E_a_), ventricular elastance (E_es_), ventriculo-arterial coupling (VAC)

End-systolic pressure (ESP) was estimated as 0.90 × systolic blood pressure (SBP), obtained by manual blood pressure cuff measurement. Stroke volume (SV) was measured from the left ventricular (LV) outflow tract (LVOT) diameter and the pulse wave Doppler signal. Left ventricular ejection fraction (LVEF) was measured using the biplane modified Simpson’s rule. Arterial elastance (*E*_*a*_) was thus defined as the ratio of ESP/SV by using the following formula:

$${E}_{a}=\left(\mathrm{SBP}\times 0.9\right)/\mathrm{SV}$$

Ventricular elastance (*E*_*es*_) was determined using a modified single-beat algorithm described by Chen et al. using arm cuff pressures, echocardiography-derived Doppler SV, and several timing intervals (pre-ejection time, total ejection time) [11, 12]. More in detail, *E*_*es*_ was calculated by the formula:

$${E}_{es}=\left[\mathrm{DBP}-\left({\mathrm{E}}_{\mathrm{Nd}}\left(\mathrm{est}\right)\times \mathrm{SBP}\times 0.9\right)\right]/{\mathrm{E}}_{\mathrm{Nd}}\left(\mathrm{est}\right)\times \mathrm{SV}$$

where DBP is diastolic arm-cuff blood pressure and E_Nd_(est) is the estimated normalized ventricular elastance at the onset of ejection calculated as follows:

$${E}_{Nd}\left(est\right)=0.0275-0.165\times \mathrm{LVEF}+0.3656\times \left(\mathrm{DBP}/\left(\mathrm{SBP}\times 0.9\right)\right)+0.515\times {\mathrm{E}}_{\mathrm{Nd}}\left(\mathrm{avg}\right)$$

where E_Nd_(avg) is the averaged normalized ventricular elastance at the onset of the ejection obtained by the formula:

$${E}_{Nd}\left(avg\right)=0.35695-7.2266\times \mathrm{tNd}+74.249\times {\mathrm{tNd}}^{\wedge }2-307.39\times {\mathrm{tNd}}^{\wedge }3+684.54\times {\mathrm{tNd}}^{\wedge }4-856.92\times {\mathrm{tNd}}^{\wedge }5+571.95\times {\mathrm{tNd}}^{\wedge }6-159.1\times {\mathrm{tNd}}^{\wedge }7$$

where tNd represents the ratio of the pre-ejection period to the total systolic period measured on the aortic pulse Doppler [11, 24].

VAC was estimated by the *E*_*a*_/*E*_*es*_ ratio [12]. Supplementary Fig. 1 illustrates how to assess VAC in routine practice, with all the necessary echocardiographic measurements.

Both *E*_*a*_ and *E*_*es*_ are expressed in mmHg/ml. Normal invasively determined *E*_*a*_ and *E*_*es*_ in resting subjects are 2.2 ± 0.8 mmHg/ml and 2.3 ± 1.0 mmHg/ml, respectively [13]. For maximal cardiac work and efficiency, the coupling ratio of *E*_*a*_/*E*_*es*_ resides between 0.5 and 1.2 [13]. In failing hearts, this ratio increases as cardiac function declines and arterial load increases (i.e., ventriculo-arterial uncoupling) [12].

Stored images and loops were reviewed by a second blinded experienced operator (V.A.DS.) who repeated the following measurements: SV, ventricular volumes with LVEF.

### Statistical analysis

Statistical analyses were performed using STATA 18 software (StataCorp, College Station, USA). All variables were tested for normality by Shapiro–Wilk test. Results were expressed as mean ± SD (or median and IQR) for continuous variables, or as number of cases and percentage for categorical variables. The Student’s *t*-test and Mann–Whitney *U* test or the χ-square test were used to compare the differences between two groups. One-way analysis of variance (ANOVA) or the Kruskal–Wallis tests were used to test the differences between more than two groups. To assess the differences of clinical, laboratory and instrumental variables over a follow-up period of 6 months, the McNemar test and the Student’s *t*-test for paired samples or the Wilcoxon matched-pairs signed-rank test were used.

The relationship between VAC, its components, and NT-proBNP and transthyretin levels were analyzed, and Spearman’s (ρ) correlation coefficients obtained. *P* < 0.05 values were considered statistically significant.

Interobserver reliability for echocardiographic measurements was assessed by Intraclass Correlation Coefficient (ICC) with cut-off values > 0.7, > 0.8 and > 0.9 indicating good, optimal and excellent agreement, respectively (Supplementary Table 1).

Univariate and multivariate logistic regression analyses were performed to assess independent associations between variables with potential pathophysiological/clinical relevance and a high NAC/Mondor stage.

## Results

### Study population

The main clinical, laboratory and instrumental features of the 114 participants are detailed in Table 1. Median age was 79 (interquartile range [IQR]: 73–84) years, and the majority (89%) were men.

**Table 1:** Overall clinical, laboratory, ECG and echocardiographic features of the study cohort

	*n* = 114
*Men, n (%)*	102 (89)
*Age, (years)*	79 (73–84)
*Height, (cm)*	172 (165–175)
*Weight, (kg)*	75 (66–83)
*Body surface area—BSA, (m*^*2*^*)*	1.88 ± 0.21
*Arterial hypertension, n (%)*	85 (75)
*Diabetes mellitus, n (%)*	19 (17)
*Dyslipidemia, n (%)*	53 (46)
*Active smoker, n (%)*	4 (4)
*Coronary artery disease, n (%)*	24 (21)
*Previous PCI/CABG, n (%)*	18 (16)
*Aortic stenosis at baseline, n (%)*	6 (5)
*Aortic stenosis at follow-up, n (%)*	3 (3)
*Previous TAVI/AVR, n (%)*	3 (3)
*Atrial fibrillation, n (%)*	
*No*	63 (55)
*Yes, history*	25 (22)
*Yes, permanent*	26 (23)
*Cardiac implantable electronic devices at baseline, n (%)*	
*No*	95 (83)
*Pacemaker*	12 (10)
*ICD*	4 (4)
*CRT*	3 (3)
*Heart failure history (prior to baseline diagnosis), n (%)*	54 (47)
*Carpal tunnel syndrome, n (%)*	74 (65)
*Polineuropathy symptoms, n (%)*	19 (17)
*Cancer, n (%)*	26 (23)
BNP, (ng/l)^*^	382 (201–491)
NT-proBNP, (ng/l)^*^	3144 (1203–5555) over 110 observations
*Serum creatinine, (mg/dl)*	1.19 (1–1.55)
*eGFR, (ml/min/1.73m*^*2*^*)*	42 (31–55)
NAC/Mondor stage, n (%)^*^	
*I*	34 (31)
*II*	32 (29)
*III*	43 (39) over 109 observations
*High sensitivity troponin I—hsTnI, (ng/l)*	42 (26–74)
*Transthyretin (TTR) levels, (mg/dl)*	30 (26–34)
*Systolic blood pressure—SBP, (mmHg)*	126 ± 19
*Diastolic blood pressure -DBP, (mmHg)*	79 ± 10
*New York Heart Association (NYHA) functional class, n (%)*	
*I*	8 (7)
*II*	101 (89)
*III*	5 (4)
*IV*	0 (0)
*Six-minute walk distance—6MWD, (mt)*	316 ± 107
*Kansas City Cardiomyopathy questionnaire—KCCQ, (score)*	74.3 (52.9–85.4)
*Tafamidis treatment, n (%)*	114 (100)
*Tafamidis treatment duration, (months)*	19 ± 8
*Furosemide, n (%)*	78 (68)
*Furosemide daily dose, (mg/24h)*	37.5 (25–62.5)
*Other diuretics, n (%)*	
*No*	99 (88)
*Hydrochlorothiazide*	5 (4)
*Torasemide*	6 (5)
*Metolazone*	1 (1)
*Indapamide*	0 (0)
*Others (e.g. bumetanide)*	0 (0)
*Acetazolamide*	1 (1)
*MRA, n (%)*	55 (49)
*MRA type, n (%)*	
*Spironolactone*	20 (36)
*Potassium canrenoate*	30 (53)
*Eplerenone*	5 (9)
*SGLT2i, n (%)*	31 (27)
*SGLT2i daily dose, (mg/24h)*	10 (10–10)
*Beta-blockers, n (%)*	71 (62)
*Beta-blocker type, n (%)*	
*Bisoprolol*	50 (70)
*Metoprolol*	14 (20)
*Carvedilol*	1 (1)
*Nebivolol*	3 (4)
*Atenolol*	1 (1)
*Propranolol*	0 (0)
*Nadolol*	0 (0)
*Sotalol*	2 (3)
*ACE-i, n (%)*	24 (21)
*ACE-i type, n (%)*	
*Ramipril*	13 (54)
*Enalapril*	4 (17)
*Perindopril*	2 (8)
*Lisinopril*	3 (13)
*Zofenopril*	2 (8)
*Others*	0 (0)
*ARB, n (%)*	17 (15)
*ARB type, n (%)*	
*Valsartan*	5 (29)
*Irbesartan*	1 (6)
*Telmisartan*	3 (18)
*Losartan*	1 (6)
*Olmesartan*	3 (18)
*Candesartan*	4 (24)
*ARNi, n (%)*	8 (7)
*Calcium channel blockers, n (%)*	7 (6)
*Calcium channel blockers type, n (%)*	
*Amlodipine*	5 (71)
*Barnidipine*	1 (14)
*Lacidipine*	0 (0)
*Lercanidipine*	1 (14)
*Manodipine*	0 (0)
*Verapamil*	0 (0)
*Diltiazem*	0 (0)
*Amiodarone, n (%)*	12 (11)
*Digoxin, n (%)*	1 (1)
*Antiarrhythmics (Ic class), n (%)*	0 (0)
*Ivabradine, n (%)*	0 (0)
*Ranolazine, n (%)*	1 (1)
*Nitrates, n (%)*	0 (0)
*Aspirin, n (%)*	15 (13)
*Other antiplatelet agents, n (%)*	6 (5)
*DOAC, n (%)*	65 (57)
*aVK, n (%)*	4 (4)
*Lipid lowering agents, n (%)*	48 (42)
*Rhythm, n (%)*	
*Sinus*	47 (41)
*Atrial fibrillation (AF)*	36 (32)
*Atrial flutter*	6 (5)
*Atrial and ventricular paced*	5 (4)
*Ventricular paced with underlying AF*	15 (13)
*Alternance spontaneous QRS and paced QRS*	0 (0)
*Ventricular paced with spontaneous atrium (VDD)*	5 (4)
*Atrial paced with spontaneous QRS*	0 (0)
*Atrial ectopic beats, n (%)*	7 (6)
*Ventricular ectopic beats, n (%)*	21 (18)
*Heart rate, (bpm)*	68 (60–80)
*PR interval, (msec)*	210 (180–240)
*First degree AV block, n (%)*	25 (22)
*Mobitz I AV block, n (%)*	0 (0)
*Left anterior fascicular block, n (%)*	38 (33)
*Left posterior fascicular block, n (%)*	2 (2)
*Right bundle branch block, n (%)*	18 (16)
*Left bundle branch block, n (%)*	5 (4)
*QRS duration*	138 ± 30
*Low QRS voltages, n (%)*	28 (25)
*Peripheral QRS score, (mm)*	30 (26–41)
*Left ventricular hypertrophy—LVH, n (%)*	1 (1)
*Pseudonecrosis, n (%)*	11 (10)
*T wave inversion—TWI, n (%)*	1 (1)
*Repolarization abnormalities, n (%)*	1 (1)
*Interventricular septum thickness, (mm)*	18 (16–20)
*Posterior wall thickness, (mm)*	15 (13–17)
*End-diastolic diameter, (mm)*	45 (42–49)
*End-diastolic volume, (ml)*	98 (80–118)
*End-systolic volume, (ml)*	46 (34–61)
*Left ventricular ejection fraction, (%)*	52 (42–59)
*Stroke volume (Doppler method), (ml)*	60 (48–76)
*Cardiac output (stroke volume* × *heart rate), (l/min)*	4.42 ± 1.42
*Cardiac index (cardiac output/BSA), (l/min/m*^*2*^*)*	2.35 ± 0.72
*Mitral E velocity, (m/s)*	0.78 ± 0.20
*E/A ratio*	1.18 (0.73–2.14)
*e’ septal velocity, (m/s)*	0.04 (0.04–0.05)
*e’ lateral velocity, (m/s)*	0.06 (0.05–0.07)
*E/e’ ratio (average)*	14.5 (11.9–18.3)
*Left atrial area, (cm*^*2*^*)*	26 ± 4
*Left atrial volume indexed for BSA, (ml/m*^*2*^*)*	50 (41–60)
*TAPSE, (mm)*	17 ± 5
*PASP, (mmHg)*	44 ± 11 (over 26 observations)
*Ventricular elastance—E*_*es*_*, (mmHg/ml)*	1.48 (1.22–1.84)
*Arterial elastance—E*_*a*_*, (mmHg/ml)*	1.86 (1.49–2.29)
*Ventriculo-arterial coupling—E*_*a*_*/E*_*es*_	1.24 (0.96–1.58)

*PCI* percutaneous coronary intervention, *CABG* coronary artery bypass graft, *TAVI* transcatheter aortic valve implantation, *AVR* aortic valve replacement, *ICD* implantable cardioverter defibrillator, *CRT* cardiac resynchronization therapy, *eGFR* estimated glomerular filtration rate, *NAC* National Amyloidosis Centre (stage I defined as NT-proBNP < 3000 ng/l and eGFR > 45 ml/min/1.73 m^2^; stage III defined as NT-proBNP > 3000 ng/l and eGFR < 45/min/.1.73 m^2^; stage II the remainder); *MRAs*, mineralocorticoid receptor antagonists; *SGLT2i*, sodium glucose cotransporters-2 inhibitors; *ACEi*, angiotensin-converting enzyme inhibitors; *ARB*, angiotensin receptor blockers; *ARNi*, angiotensin receptor-neprilysin inhibitors; *DOAC*, direct oral anticoagulants; *aVK*, vitamin K antagonist anticoagulants; *TAPSE*, tricuspid annular plane systolic excursion; *PASP*, pulmonary artery systolic pressure

^*^In patients with more severe chronic kidney disease (CKD), circulating levels of BNP together of instead of NT-proBNP were measured (the first being considered independent of eGFR compared to NT-proBNP). By consequence NAC/Mondor stage was not available in 5 patients

Mean systolic blood pressure was 126 ± 19 mmHg. Median NT-proBNP was 3144 (1203–5555) ng/l, and median eGFR was 42 (31–55) ml/min/1.73 m^2^. More than two-thirds of patients were classified in a stage II or III according to NAC/Mondor. Conventional supporting HF treatments were commonly prescribed. Sixty-eight percent of patients were treated with furosemide, with a median daily dose of 37.5 (25–62.5) mg/24 h. Beta-blockers and mineralocorticoid receptor antagonists (MRAs) were also frequently prescribed (in 62% and 49% of the entire cohort, respectively), while angiotensin-converting enzyme inhibitors (ACEi), ARB — angiotensin receptor blockers (ARB), angiotensin receptor-neprilysin inhibitors (ARNi) and calcium-channel blockers were much less frequently prescribed (in 21%, 15%, 7%, and 6% of the entire cohort, respectively). The more recently introduced sodium glucose cotransporters-2 inhibitors (SGLT2i) were prescribed in 27% of the patients.

Only 41% of the patients were in sinus rhythm, and atrial fibrillation (AF) was present in almost one-third (32%) of the entire cohort. A first-degree atrioventricular block was present in 22% of the patients, with a median PR interval in the entire cohort of 210 (180–240) msec. In terms of ventricular conduction disturbances, left anterior fascicular block and right bundle branch block in isolation or combination were more frequent than a left bundle branch block morphology. Mean QRS duration was 138 ± 30 ms. Low QRS voltages were present in 25% of the entire cohort.

In terms of echocardiographic features, median interventricular septum thickness was 18 (16–20) mm, with a median LVEF of 52 (42–59)%.

### Left ventricular-arterial coupling (VAC) and its components in the study population

Data is shown in Table 2. Median *E*_*a*_ was 1.86 (1.49–2.29) mmHg/ml, median *E*_*es*_ 1.48 (1.22–1.84) mmHg/ml, median VAC 1.24 (0.96–1.58). Patients were also split into two groups according to preexisting literature reporting cut-off values for VAC > 1 or > 1.3 (Table 2b) [9, 10]. According to the first proposed cut-off value, 78 (68%) patients had a VAC > 1 with a median value of 1.43 (1.21–1.67) *vs* 36 (32%) with the ratio < 1 and a median value of 0.91 (0.81–0.95). Fifty-four (47%) patients had a VAC > 1.3 (1.58[1.41–1.75] *vs* 0.97[0.90–1.10] of those with the ratio < 1.3). The entire cohort was also split into tertiles, and the cut-offs with the minimum and maximum values are reported in Table 2c.

**Table 2:** Left ventricular elastance, aortic elastance and ventriculo-arterial coupling in the study cohort

**a**			
			**Median value and IQR**
Ventricular elastance—E_es_, (mmHg/ml)			1.48 (1.22–1.84)
Arterial elastance—E_a_, (mmHg/ml)			1.86 (1.49–2.29)
Ventriculo-arterial coupling—E_a_/E_es_			1.24 (0.96–1.58)
**b**			
		**n (%)**	**Median value and IQR**
Ventriculo-arterial coupling—E_a_/E_es_ > 1		78 (68)—yes vs 36 (32)—no	1.43 (1.21–1.67) vs 0.91 (0.81–0.95)
Ventriculo-arterial coupling—E_a_/E_es_ > 1.3		54 (47)—yes vs 60 (53)—no	1.58 (1.41–1.75) vs 0.97 (0.90–1.10)
**c**			
	**1**^**st**^ **tertile** **n = 38**	**2**^**nd**^** tertile** **n = 38**	**3**^**rd**^ **tertile** **n = 38**
Ventricular elastance—E_es_, (mmHg/ml)	0.59–1.29* 1.13 (1.05–1.22)	1.32–1.66* 1.48 (1.40–1.56)	1.67–3.71* 2.01 (1.84–2.37)
Arterial elastance—E_a_, (mmHg/ml)	0.85–1.60* 1.43 (1.24–1.49)	1.6–2.0* 1.86 (1.75–1.95)	2.06–3.3* 2.6 (2.29–2.87)
Ventriculo-arterial coupling—E_a_/E_es_	0.6–1.06* 0.92 (0.82–0.96)	1.06–1.43* 1.24 (1.11–1.35)	1.43–2.4* 1.68 (1.58–1.78)

^*^Min and max value

### Arterial and ventricular elastance and their ratio (VAC) and NAC/Mondor stage

The results are reported in Table 3 and Table 4.

**Table 3:** Summary statistics for left ventricular elastance, aortic elastance and ventriculo-arterial coupling by NAC stage

	NAC I *n* = 34 Median and IQR	NAC II *n* = 32 Median and IQR	NAC III *n* = 43 Median and IQR	***p* value**
*Ventricular elastance—E*_*es*_*, (mmHg/ml)*	1.52 (1.32–1.89)	1.49 (1.24–1.83)	1.39 (1.17–1.92)	0.636
*Arterial elastance—E*_*a*_*, (mmHg/ml)*	1.61 (1.28–1.87)	2.05 (1.60–2.72)	1.89 (1.53–2.4)	0.0065
*Ventriculo-arterial coupling—E*_*a*_*/E*_*es*_	1.02 (0.89–1.30)	1.35 (1.04–1.66)	1.41 (1–1.65)	0.0003

**Table 4:** Summary statistics for left ventricular elastance, aortic elastance and ventriculo-arterial coupling by NAC stage (NAC I vs II/III)

	NAC I *n* = 34 Median and IQR	NAC II/III *n* = 75 Median and IQR	*p* value
*Ventricular elastance—E*_*es*_*, (mmHg/ml)*	1.52 (1.32–1.89)	1.46 (1.17–1.85)	0.396
*Arterial elastance—E*_*a*_*, (mmHg/ml)*	1.61 (1.28–1.87)	1.95 (1.55–2.48)	0.002
*Ventriculo-arterial coupling—E*_*a*_*/E*_*es*_	1.02 (0.89–1.30)	1.38 (1–1.65)	< 0.001

### Associations between echocardiographic VAC and hemodynamic congestion

**Fig. 1:**
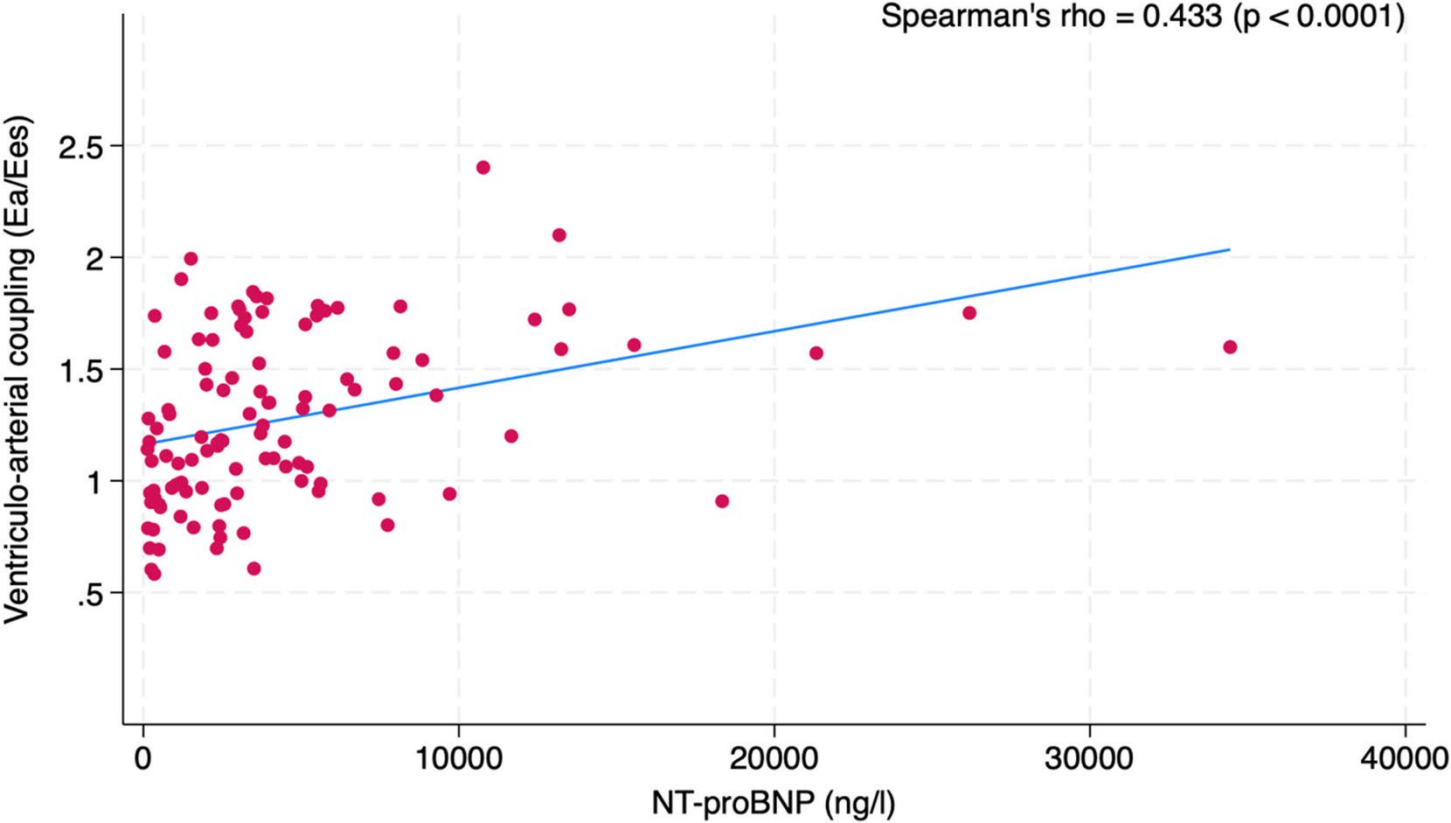
Association between the *E*_*a*_ (arterial elastance)*/E*_*es*_ (ventricular elastance) ratio (ventriculo-arterial coupling—VAC) and N-terminal pro-B-type natriuretic peptide (NT-proBNP) levels

We determined the relationships between the echocardiographic-derived measurement of VAC and a commonly accepted measurement of HF severity and hemodynamic congestion: plasma NT-proBNP levels. We found a moderate statistically significant correlation (ρ = 0.433; *p* < 0.0001) between VAC and NT-proBNP levels (Fig. 1). Interestingly, when considering the single components of the ratio (i.e., *E*_*a*_ and *E*_*es*_) this correlation became much weaker, although remaining statistically significant (ρ = 0.238; *p* = 0.012 for *E*_*a*_ and ρ = − 0.20: *p* = 0.036 for *E*_*es*_, respectively).

In contrast, serum TTR levels, currently used in clinical practice as an ancillary parameter to monitor treatment response and disease progression, did not show any correlation with the echocardiographic-derived measurement of VAC (ρ = 0.107; *p* = 0.325) [25].

### Main clinical, laboratory and instrumental features of patients according to the tertile of VAC

These results are shown in detail in Table 5. Patients in the third (upper) tertile showed overall worse clinical, laboratory and instrumental features compared with those in the first and second tertile. More in detail, they showed higher plasma NT-proBNP levels and lower eGFR. This was accompanied by a higher percentage of patients with a NAC/Mondor stage II or III among those in the third tertile, with only 6% being classified as NAC/Mondor stage I. High sensitivity troponin-I levels were also significantly higher in the subgroup of patients in the third tertile. Interestingly, we did not find any significant difference in terms of disease progression (defined accordingly to the recently proposed parameters) compared to previous (i.e., six months before) visit [26]. Patients in the third tertile were more frequently treated with MRAs, SGLT2i, and beta-blockers. Regarding ECG features, atrial fibrillation was much less common in patients in the third tertile (this could be worth noting because plasma NT-proBNP levels are influenced and are usually higher in patients with this arrhythmia). We did not find other significant differences in terms of ECG features. The analysis of echocardiographic features revealed significantly worse indexes of global LV systolic function (i.e., LVEF and Doppler-derived stroke volume). Arterial elastance (*E*_*a*_) was significantly higher in the third tertile (2.16[1.86–2.75] mmHg/ml), suggesting an increased afterload imposed to the LV despite the lack of significant differences in terms of both age and systolic blood pressure (both potential determinants of increased afterload and arterial stiffness). Concomitant to increased *E*_*a*_, ventricular elastance (*E*_*es*_) was significantly lower in patients in the third tertile (1.25[1.11–1.46] mmHg/ml). Finally, there was an overall ventriculo-arterial uncoupling in these patients, as suggested by a median VAC equal to 1.68 (1.58–1.78). It might be relevant to note that the prevalence of arterial hypertension was similar in the three tertiles.

**Table 5:** Main clinical, laboratory and instrumental features of patients according to the tertile of ventriculo-arterial coupling (VAC)

	1 st tertile *n* = 38	2nd tertile *n* = 38	3rd tertile *n* = 38	*p* value
*Men, n (%)*	33 (87)	33 (87)	36 (95)	0.432
*Age, (years)*	79 (73–83)	79 (73–83)	81 (76–85)	0.247
*Arterial hypertension, n (%)*	28 (74)	30 (79)	27 (71)	0.799
*Diabetes, n (%)*	7 (18)	2 (5)	10 (26)	**0.036**
*Atrial fibrillation, n (%)*				0.964
*No*	22 (58)	22 (58)	19 (50)	
*Yes, history*	8 (21)	8 (21)	9 (24)	
*Yes, permanent*	8 (21)	8 (21)	10 (26)	
*Systolic blood pressure, (mmHg)*	134 ± 17	126 ± 19	119 ± 19	0.820
*Diastolic blood pressure, (mmHg)*	82 ± 10	79 ± 9	77 ± 9	0.502
*NT-proBNP, (ng/l)*	2492 (492–4225)	2547 (1249–4996)	4898 (3017–8424)	**0.001**
*Serum creatinine, (mg/dl)*	1.1 (0.97–1.36)	1.1 (0.89–1.55)	1.41 (1.13–1.91)	**0.004**
*eGFR, (ml/min/1.73 mq)*	47 (37–56)	48 (31–61)	35 (24–44)	**0.003**
*NAC/Mondor stage, n (%)*				**0.001**
*I*	18 (47)	14 (39)	2 (6)	
*II*	8 (21)	10 (28)	14 (40)	
*III*	12 (32)	12 (33)	19 (54)	
*High sensitivity troponin I, (ng/l)*	33 (21–54)	39 (25–81)	57 (38–94)	**0.005**
*NYHA class*				0.238
*I*	4 (11)	2 (5)	2 (5)	
*II*	34 (89)	35 92)	32 (84)	
*III*	0 (0)	1 (3)	4 (11)	
*IV*	0 (0)	0 (0)	0 (0)	
*6MWD, (mt)*	331 ± 106	329 ± 99	287 ± 114	0.744
*Previous visit NT-proBNP, (ng/l)*	1860 (503–3507)	2407 (1208–4401)	3709 (2812–8840)	**0.0002**
*NT-proBNP increase (over 6 months)* > *700 ng/l and* > *30%*	6 (16)	8 (22)	8 (22)	0.871
*Furosemide at previous visit, n (%)*	20 (56)	26 (68)	30 (79)	0.098
*Furosemide dose at previous visit, (mg/24h)*	31 (25–50)	25 (25–50)	50 (25–80)	0.254
*ODI, n (%)*	7 (18)	4 (11)	4 (11)	0.645
*Progression (NT-proBNP increase* + *ODI), n (%)*	2 (5)	2 (5)	0 (0)	0.544
*Furosemide, n (%)*	22 (58)	26 (68)	30 (79)	0.159
*Furosemide daily dose, (mg/24h)*	37.5 (25–50)	25 (25–50)	37.5 (12.5–80)	0.879
*MRA, n (%)*	14 (37)	17 (45)	24 (65)	**0.048**
*SGLT2i, n (%)*	4 (11)	10 (26)	16 (42)	**0.006**
*Beta-blockers, n (%)*	20 (53)	20 (53)	31 (82)	**0.012**
*ACE-I, n (%)*	8 (21)	11 (29)	5 (13)	0.263
*ARB, n (%)*	6 (16)	6 (16)	5 (13)	1.000
*ARNi, n (%)*	0 (0)	4 (11)	4 (11)	0.124
*Calcium-channel blockers, n (%)*	3 (8)	3 (8)	1 (3)	0.696
*Amiodarone, n (%)*	3 (8)	3 (8)	6 (16)	0.588
*Digoxin, n (%)*	0 (0)	1 (3)	0 (0)	1.000
*Atrial fibrillation, n (%)*	14 (37)	16 (42)	6 (16)	**0.032**
*Heart rate, (bpm)*	68 (57–80)	70 (60–80)	67 (62–80)	0.656
*PR interval, (msec)*	200 (170–230)	215 (180–245)	220 (200–245)	0.232
*Left anterior fascicular block, n (%)*	11 (29)	16 (42)	11 (29)	0.418
*Right bundle branch block, n (%)*	7 (18)	4 (11)	7 (18)	0.593
*Left bundle branch block, n (%)*	1 (3)	2 (5)	2 (5)	1.000
*Low QRS voltages, n (%)*	11 (29)	12 (32)	5 (13)	0.153
*Peripheral QRS, score, (mm)*	29.5 (27–39.5)	29 (26–39)	37 (24.5–49)	0.633
*Interventricular septum thickness, (mm)*	17 ± 2	18 ± 2	18 ± 2	0.834
*Posterior wall thickness, (mm)*	15 ± 2	15 ± 2	15 ± 3	0.203
*Left ventricular ejection fraction, (%)*	59 (54–63)	53 (45–59)	41 (38–48)	**0.0001**
*Stroke volume (Doppler method), (ml)*	73 (58–81)	62 (52–75)	47 (42–58)	**0.0001**
*Cardiac output (stroke volume* × *heart rate), (l/min)*	4.92 ± 1.59	4.60 ± 1.15	3.75 ± 1.23	**0.0007**
*Cardiac index (cardiac output/BSA), (l/min/m*^*2*^*)*	2.56 ± 0.79	2.47 ± 0.60	2.02 ± 0.64	**0.0016**
*Mitral E velocity, (m/s)*	0.86 ± 0.18	0.78 ± 0.21	0.69 ± 0.17	0.441
*Mitral E/A ratio*	1.21 (1.06–2.06)	1.07 (0.62–2.26)	0.95 (0.69–1.83)	0.413
*E/e’ ratio*	14.2 (11.2–18.3)	14.9 (11.8–20.14)	14.1 (12.6–17)	0.791
*Left atrial volume indexed for BSA, (ml/m2)*	46 (38–55)	52 (43–62)	53 (44–60)	0.076
*Ventricular elastance—E*_*es*_* (mmHg/ml)*	1.78 (1.56–2.34)	1.40 (1.16–1.67)	1.25 (1.11–1.46)	**0.0001**
*Arterial elastance—E*_*a*_* (mmHg/ml)*	1.56 (1.45–2.03)	1.72 (1.48–1.97)	2.16 (1.86–2.75)	**0.0002**
*Ventriculo-arterial coupling—E*_*a*_*/E*_*es*_	0.92 (0.82–0.96)	1.24 (1.11–1.35)	1.68 (1.58–1.78)	**0.0001**

*eGFR* estimated glomerular filtration rate, *NAC* National Amyloidosis Centre (stage I defined as NT-proBNP < 3000 ng/l and eGFR > 45 ml/min/1.73m^2^; stage III defined as NT-proBNP > 3000 ng/l and eGFR < 45/min/1.73m^2^; stage II the remainder), *NYHA* New York Heart Association functional class, *6MWD* 6-min walk distance, *ODI* outpatient diuretic intensification, *MRAs* mineralocorticoid receptor antagonists, *SGLT2i* sodium glucose cotransporters-2 inhibitors, *ACEi* angiotensin-converting enzyme inhibitors, *ARB* angiotensin receptor blockers, *ARNi* angiotensin receptor-neprilysin inhibitors

### Independent predictors of a high NAC/Mondor stage

To test the hypothesis that ventriculo-arterial uncoupling could be an independent determinant of a high disease stage (i.e., II–III according to the NAC/Mondor staging system), univariate and multivariate logistic regression analyses were carried out to assess the role of this and other variables with potential pathophysiological/clinical relevance. The results are shown in Table 6. VAC remained a significant independent determinant of a high NAC/Mondor stage both in the univariate analysis (OR[95% CI] = 15.39[3.51–67.35], *p* < 0.001) and in a multivariate model (OR[95% CI] = 11.26 [1.98–63.81], *p* = 0.006) which also included age (OR[95% CI] = 1.11[1.04–1.19], *p* = 0.003) and furosemide treatment (OR[95% CI] = 4.60[1.62–13.02], *p* = 0.004).

**Table 6:** Univariate and multivariate regression analysis to assess the independent predictors of a high NAC/Mondor stage II/III

Variables	Unadjusted Odds Ratio (95% Confidence Interval)	*p-*value	Multivariate Adjusted Odds Ratio (95% Confidence Interval)	*p-*value
Age, (years)	1.13 (1.06–1.20)	** < 0.001**	1.11 (1.04–1.19)	**0.003**
Systolic blood pressure, (mmHg)	0.96 (0.93–0.98)	**0.001**	Not included in the model**	-
Tafamidis treatment duration, (months)	1.02 (0.96–1.07)	0.545	-	-
Furosemide treatment, (no/yes)	6.22 (2.54–15.26)	** < 0.001**	4.60 (1.62–13.02)	**0.004**
Furosemide daily dose, (mg/24 h)	1.02 (0.99–1.04)	0.158	-	-
MRA, (no/yes)	5.78 (2.23–14.97)	** < 0.001**	Not included in the model	-
ACEi, (no/yes)	0.69 (0.27–1.79)	0.451	-	-
SGLT2i, (no/yes)	5.17 (1.44–18.56)	**0.012**	Not included in the model	-
Beta-blockers, (no/yes)	1.58 (0.69–3.59)	0.275	-	-
Atrial fibrillation, (no/yes)	1.53 (0.60–3.87)	0.370	-	-
QRS duration, (msec)	1.05 (1.02–1.08)	**0.003**	Not included in the model	-
IVSd, (mm)	1.08 (0.90–1.29)	0.414	-	-
Left ventricular ejection fraction, (%)	0.89 (0.84–0.94)	** < 0.001**	Not included in the model**	-
LAVi, (ml/m2)	1.05 (1.01–1.10)	**0.014**	Not included in the model	-
E/e’ (average)	1.09 (0.99–1.20)	0.070	-	-
Ventricular elastance—*E*_*es*_	0.83 (0.42–1.66)	0.602	-	-
Arterial elastance—*E*_*a*_	3.72 (1.53–9.05)	**0.004**	Not included in the model**	-
Ventriculo-arterial coupling (VAC)	15.39 (3.51–67.35)	** < 0.001**	11.26 (1.98–63.81)	**0.006**

*NAC* (National Amyloidosis Centre)/Mondor staging system (stage I defined as NT-proBNP < 3000 ng/l and eGFR > 45 ml/min/1.73 m^2^; stage III defined as NT-proBNP > 3000 ng/l and eGFR < 45/min/1.73 m^2^; stage II the remainder): *MRAs*, mineralocorticoid receptor antagonists; *ACEi*, angiotensin-converting enzyme inhibitors; *SGLT2i*, sodium glucose cotransporters-2 inhibitors; *IVSd*, interventricular septum thickness, diastole (mm); *LAVi*, left atrial volume indexed for body surface area

^**^Variables not included in the model due to potential collinearity with VAC (as components of *E*_*es*_ and *E*_*a*_)

^*^Model A (presented in the table): 109 patients were computed in a multivariate model including the following variables: age, furosemide treatment (no/yes), ventriculo-arterial coupling (VAC)

## Discussion

ATTRwt-CM is characterized by complex pathophysiology and hemodynamic adaptations [1]. This in turn makes the clinical and pharmacological management of the patients extremely challenging, and affects the progression and overall prognosis of the disease [1]. In this setting, simple parameters derived from clinical features and echocardiographic measurements able to catch both LV and vascular hemodynamic adaptations, could act as reasonable prognosticators refining patients’ risk and disease status, thus helping clinicians in the management of HF supportive therapies. To the best of our knowledge, this is the first study specifically aimed to investigate the role of left VAC in ATTRwt-CM.

Our findings, deriving from a contemporary cohort of patients, suggest the following: (1) The noninvasively determined *E*_*a*_/*E*_*es*_ ratio which defines VAC is significantly higher in patients with NAC/Mondor stages II and III suggesting ventriculo-arterial uncoupling in these individuals; (2) VAC is associated with plasma NT-proBNP levels, routinely used in clinical practice to define hemodynamic congestion; (3) Patients with the *E*_*a*_/*E*_*es*_ in the upper tertile show a worse clinical, laboratory and instrumental profile; (4) The *E*_*a*_/*E*_*es*_ ratio is an independent determinant of a high NAC/Mondor stage at both univariate and multivariate logistic regression analysis, thus suggesting its role as a prognosticator and as useful parameter to further refine patients’ stratification.

Previous studies have shown that right ventricle-to-pulmonary artery coupling (RV-PAc) evaluated by using the tricuspid annular plane systolic excursion (TAPSE)/pulmonary artery systolic pressure (TAPSE/PASP) ratio has a definite prognostic role in patients with systemic amyloidoses with cardiac involvement, being a marker of advanced disease able to predict mortality and HF hospitalization [27, 28]. One of the main limitations of these studies was represented by the inclusion of patients with both AL and ATTR amyloidoses, which are distinct nosological entities with a largely different (and yet unexplained) cardiovascular performance, at both the systolic and the diastolic level. More recently, in a multicentre study from Germany involving 418 patients with ATTR-CM treated with tafamidis, RV-PAc ratio at first presentation emerged as a robust marker for risk stratification, and patients with the ratio ≤ 0.382 mm/mmHg exhibited significantly lower survival within 3 years of follow-up [29]. However, none of the aforementioned studies investigate the role of the “left-sided” VAC, which might also have useful practical repercussions for the management of supporting HF therapies, bearing in mind the complex hemodynamics of the disease [27–29]. Our data suggests that a higher *E*_*a*_/*E*_*es*_ ratio, a reliable marker to define ventriculo-arterial uncoupling, is more common in patients with an advanced NAC/Mondor stage. This is in line with the results of the studies investigating the role of RV-PAc [29].

Although somewhat not unexpected, another interesting finding of our study is represented by the association of the *E*_*a*_/*E*_*es*_ ratio with plasma NT-proBNP levels, a hallmark of hemodynamic congestion. Interestingly, this association was lost when considering the single components of the ratio, i.e., arterial and ventricular elastances. These findings demonstrate that the pathophysiological significance of chamber elastance alone is much less relevant than the matching of ventricular and arterial elastance [12]. In failing hearts, the *E*_*a*_/*E*_*es*_ ratio increases as cardiac function declines and arterial load increases to maintain systolic pressure [12]. This means that it is indeed the coupling of cardiac performance and arterial response to dictate the severity of the disease, giving an idea of cardio-aortic adaptation. Our data did not allow us to ascertain any potential association between tafamidis treatment and VAC and its components due to the cross-sectional nature of our study. Median treatment duration in our cohort was 18 months, and data deriving from the ATTR-ACT trial suggests that the drug is “slow-acting” with the beneficial effects (in terms of hard endpoints) becoming evident after this period of treatment [17]. We can speculate about the fact that a positive hemodynamic impact is expected to precede hard clinical endpoints; however, we should also consider the potential impact of HF supporting therapies which working in parallel might potentially act as confounders partially masking the beneficial effects of the disease-modifying drug.

In our study, patients with the *E*_*a*_/*E*_*es*_ ratio in the upper tertile were more frequently treated with MRAs, SGLT2i and beta-blockers, suggesting a more advanced HF state. It is worth noting the fact that age and systolic blood pressure did not significantly differ in this subgroup of patients, and that the prevalence of AF was also lower among these patients. In the aforementioned study from Germany investigating RV-Pac, the prevalence of AF was higher in patients with impaired TAPSE/PASP ratio [28]. However, longitudinal function (i.e., TAPSE) is negatively impacted by AF, while the arrhythmia does not seem to directly impact on the components of the *E*_*a*_/*E*_*es*_ ratio.

Our data suggests that the *E*_*a*_/*E*_*es*_ ratio is an independent determinant of a high NAC/Mondor stage. Although preliminary, according to these results we can speculate that VAC could be a useful prognosticator and that it could be used to better refine disease state and progression in patients with ATTR-CM. Unfortunately, we did not find significant differences in terms of disease progression, defined accordingly to the criteria proposed by Ioannou et al. (i.e., NT-proBNP increase > 700 ng/l and > 30% plus outpatient diuretic intensification — ODI) in patients with ventriculo-arterial uncoupling; however, we considered a 6-month follow-up instead of the 12 months of the study by Ioannou et al. [26].

## Study limitations

Our results should be interpreted in light of some limitations. First, this was a single-centre study. This, on the other hand, has probably contributed to reduce the potential sources of bias and to uniform methodology (especially when considering some echocardiographic measurements). Another limitation of the study is represented by its observational cross-sectional design, with a lack of information regarding major adverse cardiovascular events (e.g., cardiovascular death, HF hospitalizations). For the same reason, the potential role of VAC and its components on clinical decision making (i.e., changes in conventional unloading and HF therapies) cannot be established by this study. We provided a “snapshot” of the hemodynamic status of patients in different stages of their disease journey. A large multicenter prospective study will help confirm the usefulness of the echocardiographic indexes of left ventriculo-arterial coupling, as parameters useful to better define and refine disease state/stage and prognosis. Another limitation of our study is represented by the lack of information regarding the potential role of tafamidis on VAC; however, this was beyond the scope of the present study. Finally, the exclusion of patients with NYHA class III should be considered a potential limitation, since the effects of conventional HF medications (with their effects on preload, afterload, myocardial contractility) on VAC would be even more important in these subjects.

## Conclusion

In a contemporary cohort of patients with ATTRwt-CM, the noninvasively determined arterial and ventricular elastances and their ratio defining VAC are able to identify a worse and severe disease stage, independently from other commonly used parameters. Moreover, they are potentially useful for a tailored approach to HF supporting treatments in the single patient, although our study should be considered hypothesis-generating, and further research is needed to provide more definite answers.

## Clinical perspective

Although further studies are needed to specifically address this topic, the noninvasively determined arterial and ventricular elastances and their ratio defining ventriculo-arterial coupling, might potentially help optimize the pharmacological management of patients with ATTR-CM. Unloading therapies and other supporting HF drugs might be chosen based also on their effects on the components of the *E*_*a*_/*E*_*es*_ ratio. This might allow clinicians to choose different molecules also within the same pharmacological class (e.g., in case of the still debated beta-blockers, the choice between a vasodilatory vs a non-vasodilatory agent should be tailored based also on the expected effects on *E*_*a*_ and *E*_*es*_). This should be probably one of the keys to solve the conundrum of the complex hemodynamics of amyloid cardiomyopathy, bearing in mind the lessons offered by other complex settings such as the cardiogenic shock in the intensive care unit patients [14].

## Supplementary Information

Below is the link to the electronic supplementary material.

ESM 1 (PNG 3.91 MB)

High Resolution Image (TIF 805 KB)

Supplementary file2 (DOC 204 KB)

## Data Availability

Data will be available upon specific request and under specific condition of appropriate collaborative work.

## References

[1] Rapezzi C, Aimo A, Barison A, Emdin M, Porcari A, Linhart A et al (2022) Restrictive cardiomyopathy: definition and diagnosis. Eur Heart J 43(45):4679–4693. 10.1093/eurheartj/ehac543 36269634 10.1093/eurheartj/ehac543 PMC9712030

[2] Ioannou A, Massa P, Patel RK, Razvi Y, Porcari A, Rauf MU et al (2023) Conventional heart failure therapy in cardiac ATTR amyloidosis. Eur Heart J 44(31):2893–2907. 10.1093/eurheartj/ehad347 37216684 10.1093/eurheartj/ehad347 PMC10424879

[3] Suga H, Sagawa K, Shoukas AA (1973) Load independence of the instantaneous pressure-volume ratio of the canine left ventricle and effects of epinephrine and heart rate on the ratio. Circ Res 32(3):314–322. 10.1161/01.res.32.3.314 4691336 10.1161/01.res.32.3.314

[4] Suga H, Sagawa K (1974) Instantaneous pressure-volume relationships and their ratio in the excised, supported canine left ventricle. Circ Res 35(1):117–126. 10.1161/01.res.35.1.117 4841253 10.1161/01.res.35.1.117

[5] Sagawa K (1978) The ventricular pressure-volume diagram revisited. Circ Res 43(5):677–687. 10.1161/01.res.43.5.677 361275 10.1161/01.res.43.5.677

[6] Sagawa K (1981) The end-systolic pressure-volume relation of the ventricle: definition, modifications and clinical use. Circulation 63(6):1223–1227. 10.1161/01.cir.63.6.1223 7014027 10.1161/01.cir.63.6.1223

[7] Sunagawa K, Maughan WL, Burkhoff D, Sagawa K (1983) Left ventricular interaction with arterial load studied in isolated canine ventricle. Am J Physiol 245(5 Pt 1):H773-8048. 10.1152/ajpheart.1983.245.5.H773 6638199 10.1152/ajpheart.1983.245.5.H773

[8] Sunagawa K, Maughan WL, Sagawa K (1985) Optimal arterial resistance for the maximal stroke work studied in isolated canine left ventricle. Circ Res 56(4):586–595. 10.1161/01.res.56.4.586 3978773 10.1161/01.res.56.4.586

[9] Chirinos JA (2013) Ventricular-arterial coupling: invasive and non-invasive assessment. Artery Res 7(1):10.1016/j.artres.2012.12.002. 10.1016/j.artres.2012.12.002 PMC3809068 24179554

[10] De Tombe PP, Jones S, Burkhoff D, Hunter WC, Kass DA (1993) Ventricular stroke work and efficiency both remain nearly optimal despite altered vascular loading. Am J Physiol 264(6 Pt 2):H1817–H1824. 10.1152/ajpheart.1993.264.6.H1817 8322910 10.1152/ajpheart.1993.264.6.H1817

[11] Chen CH, Fetics B, Nevo E, Rochitte CE, Chiou KR, Ding PA et al (2001) Noninvasive single-beat determination of left ventricular end-systolic elastance in humans. J Am Coll Cardiol 38(7):2028–2034. 10.1016/s0735-1097(01)01651-5 11738311 10.1016/s0735-1097(01)01651-5

[12] Ky B, French B, May Khan A, Plappert T, Wang A, Chirinos JA et al (2013) Ventricular-arterial coupling, remodeling, and prognosis in chronic heart failure. J Am Coll Cardiol 62(13):1165–1172. 10.1016/j.jacc.2013.03.085 23770174 10.1016/j.jacc.2013.03.085 PMC3943424

[13] Antonini-Canterin F, Poli S, Vriz O, Pavan D, Bello VD, Nicolosi GL (2013) The ventricular-arterial coupling: from basic pathophysiology to clinical application in the echocardiography laboratory. J Cardiovasc Echogr 23(4):91–95. 10.4103/2211-4122.127408 28465893 10.4103/2211-4122.127408 PMC5353400

[14] Trambaiolo P, Bertini P, Borrelli N, Poli M, Romano S, Ferraiuolo G et al (2019) Evaluation of ventriculo-arterial coupling in ST elevation myocardial infarction with left ventricular dysfunction treated with levosimendan. Int J Cardiol 288:1–4. 10.1016/j.ijcard.2019.04.052 31056414 10.1016/j.ijcard.2019.04.052

[15] Trambaiolo P, Figliuzzi I, Salvati M, Bertini P, Brizzi G, Tocci G et al (2022) Ventriculo-arterial coupling in the intensive cardiac care unit: a non-invasive prognostic parameter. Int J Cardiol 348:85–89. 10.1016/j.ijcard.2021.12.026 34933063 10.1016/j.ijcard.2021.12.026

[16] Maurer MS, Schwartz JH, Gundapaneni B, Elliott PM, Merlini G, Waddington-Cruz M et al (2018) Tafamidis treatment for patients with transthyretin amyloid cardiomyopathy. N Engl J Med 379(11):1007–1016. 10.1056/NEJMoa1805689 30145929 10.1056/NEJMoa1805689

[17] Kittleson MM, Ruberg FL, Ambardekar AV, Brannagan TH, Cheng RK, Clarke JO et al (2023) 2023 ACC expert consensus decision pathway on comprehensive multidisciplinary care for the patient with cardiac amyloidosis: a report of the american college of cardiology solution set oversight committee. J Am Coll Cardiol 81(11):1076–1126. 10.1016/j.jacc.2022.11.022 36697326 10.1016/j.jacc.2022.11.022

[18] Milani P, Fabris F, Mussinelli R, Sanna GD, Basset M, Benvenuti P et al (2024) Delayed identification of monoclonal protein is associated with early death in isolated cardiac AL amyloidosis. Amyloid 31(3):220–225. 10.1080/13506129.2024.2374904 38989681 10.1080/13506129.2024.2374904

[19] Katzmann JA, Clark RJ, Abraham RS, Bryant S, Lymp JF, Bradwell AR et al (2002) Serum Reference Intervals and Diagnostic Ranges for Free κ and Free λ Immunoglobulin Light Chains: Relative Sensitivity for Detection of Monoclonal Light Chains. Clin Chem 48(9):1437–1444 12194920

[20] Nuvolone MU, Sanna GD, Palladini G (2025) AL or ATTR amyloidosis? Never two without three. Circulation 151(3):274–281. 10.1161/CIRCULATIONAHA.124.072460 39836759 10.1161/CIRCULATIONAHA.124.072460

[21] Gillmore JD, Damy T, Fontana M, Hutchinson M, Lachmann HJ, Martinez-Naharro A et al (2018) A new staging system for cardiac transthyretin amyloidosis. Eur Heart J 39(30):2799–2806. 10.1093/eurheartj/ehx589 29048471 10.1093/eurheartj/ehx589

[22] Martini N, Sinigiani G, De Michieli L, Mussinelli R, Perazzolo Marra M, Iliceto S et al (2024) Electrocardiographic features and rhythm disorders in cardiac amyloidosis. Trends Cardiovasc Med 34(4):257–264. 10.1016/j.tcm.2023.02.006 36841466 10.1016/j.tcm.2023.02.006

[23] Lang RM, Badano LP, Mor-Avi V, Afilalo J, Armstrong A, Ernande L et al (2015) Recommendations for cardiac chamber quantification by echocardiography in adults: an update from the american society of echocardiography and the european association of cardiovascular imaging. J Am Soc Echocardiogr 28(1):1-39.e14. 10.1016/j.echo.2014.10.003 25559473 10.1016/j.echo.2014.10.003

[24] Holm H, Magnusson M, Jujić A, Bozec E, Girerd N (2022) How to calculate ventricular-arterial coupling? Eur J Heart Fail 24(4):600–602. 10.1002/ejhf.2456 35191147 10.1002/ejhf.2456 PMC9314840

[25] Falk RH, Haddad M, Walker CR, Dorbala S, Cuddy SAM (2021) Effect of tafamidis on serum transthyretin levels in non-trial patients with transthyretin amyloid cardiomyopathy. JACC CardioOncol 3(4):580–586. 10.1016/j.jaccao.2021.08.007 34729530 10.1016/j.jaccao.2021.08.007 PMC8543137

[26] Ioannou A, Cappelli F, Emdin M, Nitsche C, Longhi S, Masri A et al (2024) Stratifying disease progression in patients with cardiac ATTR amyloidosis. J Am Coll Cardiol 83(14):1276–1291. 10.1016/j.jacc.2023.12.036 38530684 10.1016/j.jacc.2023.12.036 PMC11004588

[27] Tomasoni D, Adamo M, Porcari A, Aimo A, Bonfioli GB, Castiglione V et al (2023) Right ventricular to pulmonary artery coupling and outcome in patients with cardiac amyloidosis. Eur Heart J Cardiovasc Imaging 24(10):1405–1414. 10.1093/ehjci/jead145 37379445 10.1093/ehjci/jead145

[28] Palmiero G, Monda E, Verrillo F, Dongiglio F, Caiazza M, Rubino M et al (2023) Prevalence and clinical significance of right ventricular pulmonary arterial uncoupling in cardiac amyloidosis. Int J Cardiol 388:131147. 10.1016/j.ijcard.2023.131147 37423570 10.1016/j.ijcard.2023.131147

[29] Schwarting SK, Poledniczek M, Metodiev Y, Stolz L, Hofmann E, Hegenbart U et al (2024) RV-PA uncoupling is associated with increased mortality in transthyretin amyloid cardiomyopathy treated with tafamidis. Clin Res Cardiol. 10.1007/s00392-024-02576-239565387 10.1007/s00392-024-02576-2

