# Supplementary figures and images for: Left ventriculo-arterial coupling in a contemporary cohort of patients with wild-type transthyretin cardiac amyloidosis treated with tafamidis
Source: Clin Res Cardiol. 2025 Nov 3;115(5):811–25. doi: 10.1007/s00392-025-02727-z (PMC13083476; doi:10.1007/s00392-025-02727-z)

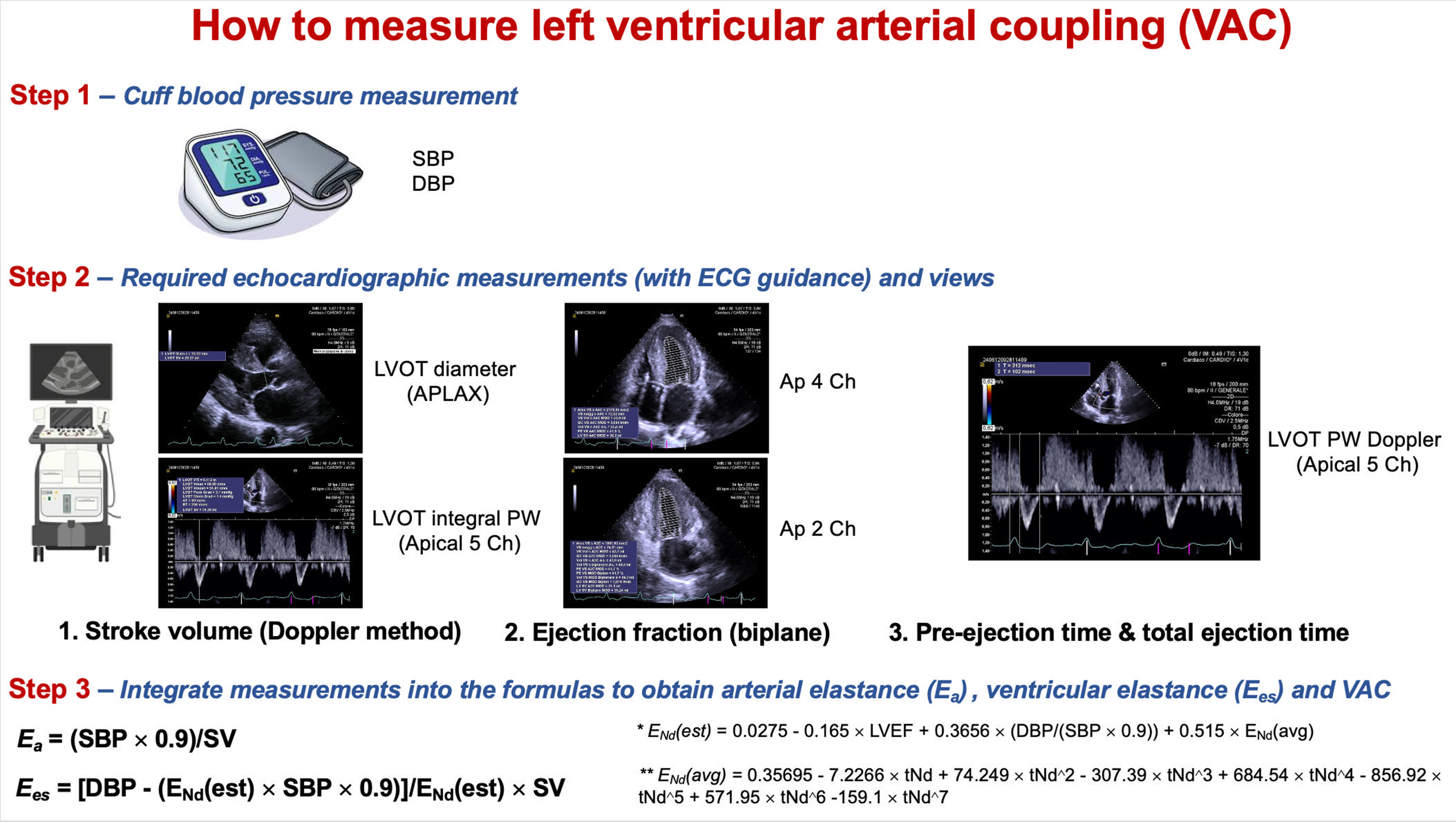

Supplement: Supplementary file 1 — (PNG 3.91 MB) [file 392_2025_2727_Fig2_ESM.png]
