# Supplementary material for: Left ventriculo-arterial coupling in a contemporary cohort of patients with wild-type transthyretin cardiac amyloidosis treated with tafamidis
Source: Clin Res Cardiol. 2025 Nov 3;115(5):811–25. doi: 10.1007/s00392-025-02727-z (PMC13083476; doi:10.1007/s00392-025-02727-z)

**Supplementary Table 1.** Intraclass Correlation Coefficient ICC (left ventricular volumes and ejection fraction)

| **Number of subjects (n)** | 114 |
| --- | --- |
| **Number of raters (k)** | 2 |
| **Model** | The same raters for all subjects.  Two-way model. |
| **Type** | Consistency |
| **Measurements** | End-diastolic volume (EDV) - Operator 1  End-diastolic volume (EDV) - Operator 2 |

**Intraclass Correlation Coefficient**

|  | **Intraclass correlation ^a^** | **95% Confidence Interval** |
| --- | --- | --- |
| **Single measures ^b^** | 0.9674 | 0.9528 to 0.9775 |
| **Average measures ^c^** | 0.9834 | 0.9758 to 0.9886 |

^a^ The degree of consistency among measurements

^b^ Estimates the reliability of single ratings.

^c^ Estimates the reliability of averages of k ratings.

________________________________________________________________________________

| **Number of subjects (n)** | 114 |
| --- | --- |
| **Number of raters (k)** | 2 |
| **Model** | The same raters for all subjects.  Two-way model. |
| **Type** | Consistency |
| **Measurements** | End-systolic volume (ESV) - Operator 1  End-systolic volume (ESV) - Operator 2 |

**Intraclass Correlation Coefficient**

|  | **Intraclass correlation ^a^** | **95% Confidence Interval** |
| --- | --- | --- |
| **Single measures ^b^** | 0.9403 | 0.9147 to 0.9584 |
| **Average measures ^c^** | 0.9692 | 0.9554 to 0.9788 |

^a^ The degree of consistency among measurements

^b^ Estimates the reliability of single ratings.

^c^ Estimates the reliability of averages of k ratings.

________________________________________________________________________________

| **Number of subjects (n)** | 114 |
| --- | --- |
| **Number of raters (k)** | 2 |
| **Model** | The same raters for all subjects.  Two-way model. |
| **Type** | Consistency |
| **Measurements** | End-systolic volume (ESV) - Operator 1  End-systolic volume (ESV) - Operator 2 |

**Intraclass Correlation Coefficient**

|  | **Intraclass correlation ^a^** | **95% Confidence Interval** |
| --- | --- | --- |
| **Single measures ^b^** | 0.9572 | 0.9382 to 0.9704 |
| **Average measures ^c^** | 0.9781 | 0.9681 to 0.9850 |

^a^ The degree of consistency among measurements

^b^ Estimates the reliability of single ratings.

^c^ Estimates the reliability of averages of k ratings.

________________________________________________________________________________

| **Number of subjects (n)** | 114 |
| --- | --- |
| **Number of raters (k)** | 2 |
| **Model** | The same raters for all subjects.  Two-way model. |
| **Type** | Consistency |
| **Measurements** | LV ejection fraction (EF) - Operator 1  LV ejection fraction (EF) - Operator 2 |

**Intraclass Correlation Coefficient**

|  | **Intraclass correlation ^a^** | **95% Confidence Interval** |
| --- | --- | --- |
| **Single measures ^b^** | 0.9403 | 0.9147 to 0.9584 |
| **Average measures ^c^** | 0.9692 | 0.9554 to 0.9788 |

^a^ The degree of consistency among measurements

^b^ Estimates the reliability of single ratings.

^c^ Estimates the reliability of averages of k ratings.

Bland Altman plot (LV EF)


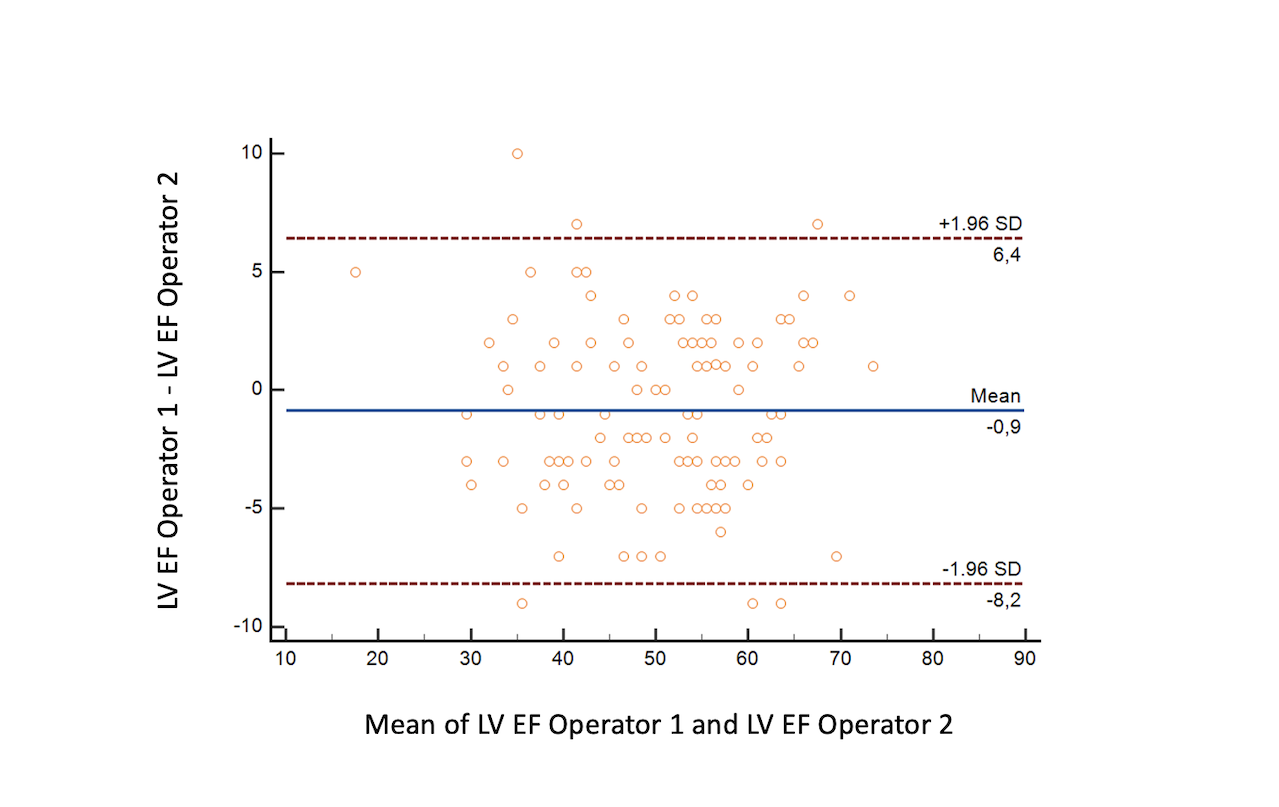

Supplement: Supplementary file 3 — Supplementary file2 (DOC 204 KB) [file 392_2025_2727_MOESM2_ESM.doc]
